# The impact of number of repeats *N* on the interlayer exchange in $[\text {Fe/MgO}]_{N}$(001) superlattices

**DOI:** 10.1038/s41598-021-81441-y

**Published:** 2021-01-21

**Authors:** Tobias Warnatz, Fridrik Magnus, Nanny Strandqvist, Sarah Sanz, Hasan Ali, Klaus Leifer, Alexei Vorobiev, Björgvin Hjörvarsson

**Affiliations:** 1 Department of Physics and Astronomy, Uppsala University, Box 516, 75120 Uppsala, Sweden; 2 Science Institute, University of Iceland, Dunhaga 3, Reykjavík, 107 Iceland; 3 Department of Materials Science and Engineering, Uppsala University, Box 534, 75121 Uppsala, Sweden; 4 Present Address: University of Konstanz, Constance, Germany

**Keywords:** Materials science, Nanoscience and technology, Physics

## Abstract

The strength of the interlayer exchange coupling in [Fe/MgO]$$_N$$(001) superlattices with 2 ≤ *N* ≤ 10 depends on the number of bilayer repeats (*N*). The exchange coupling is antiferromagnetic for all the investigated thicknesses while being nine times larger in a sample with *N* = 4 as compared to *N* = 2. The sequence of the magnetic switching in two of the samples (*N* = 4, *N* = 8) is determined using polarized neutron reflectometry. The outermost layers are shown to respond at the lowest fields, consistent with having the weakest interlayer exchange coupling. The results are consistent with the existence of quantum well states defined by the thickness of the Fe and the MgO layers as well as the number of repeats (*N*) in [Fe/MgO]$$ _{N}$$(001)superlattices.

## Introduction

Fe/MgO/Fe heterostructures are well known for their large tunnel magnetoresistance (TMR) effect^1,2^. The discovery of TMR gave rise to the development of new types of spintronics and sensing devices^3^ as well as multilevel storage schemes^4,5^. In addition to TMR, antiferromagnetic (AFM) interlayer exchange coupling (IEC) can be obtained in Fe/MgO/Fe structures with high crystalline quality^6–8^. The interlayer exchange coupling is believed to originate from spin-polarized tunneling^9–11^ through the MgO and is therefore strongly dependent on the crystalline quality of the oxide layers^12,13^. The coupling gives rise to AFM ordering of the Fe layers, unlike the oscillatory RKKY interlayer exchange coupling in metallic multilayers, and its strength decays exponentially with MgO thickness^6–8^. The coupling results in a preferred antiparallel or even perpendicular in-plane alignment of multiple adjacent magnetic layers, which opens up new possibilities for three-dimensional spintronic devices^8^. However, the experimental results continue to challenge our understanding of the fundamental principles governing interlayer exchange coupling. Recently, a sequential magnetic switching of Fe layers in interlayer exchange coupled Fe/MgO(001) superlattices was reported^14^. The observed switching of the magnetization of the Fe layers could not be rationalized using nearest neighbor interlayer interactions alone, interactions beyond nearest neighbor were needed to understand the results. Long-range interactions are not expected from current models used to describe IEC through tunnel barriers, although the effect of next nearest neighbor interactions has been observed in magnetic semiconductor multilayers^15–17^. If the IEC is extended beyond nearest neighbor, large effects on the effective interlayer exchange are expected in the few layer limit (small *N*).

Here we discuss the impact of the number of bilayer repetitions (*N*) on the exchange coupling in [Fe/MgO(001)]$_N$ superlattices. The observed changes in the hysteresis loops with *N* are used to infer the changes in the interlayer coupling. This approach has previously been used to explore the changes in the coupling in Fe/Cr multilayers^18^, where the interaction was proven to be restricted to nearest neighbor, as expected for direct exchange across antiferromagnetic layers. A very different result is obtained here: we find the coupling to be strongly influenced by the total number of repeats (*N*) in [Fe/MgO(001)]$_N$ superlattices in a non trivial way.

## Experimental details

The Fe/MgO layers were epitaxially grown via magnetron sputtering in an ultra high vacuum system with a base pressure in the low $10^{-9}$ mbar range and an operating pressure of 2.7 × 10^−3^ mbar Ar (99.999 99% purity). Prior to the deposition process, the MgO(001) substrates (10 × 10 × 1 $\text {mm}^{3}$) were annealed for 1 h at 550 °C. The substrate temperature was thereafter lowered to 165 °C, at which the Fe and the MgO layers were grown. The Fe layers (2.0 nm thick) were deposited via dc sputtering from an Fe target (99.95% purity), while the MgO layers (1.7 nm thick) were prepared via rf sputtering from a MgO target (99.99% purity). The intended repeat distance $\lambda$ is therefore 3.7 nm. The repetition number *N* of [Fe/MgO]$_N$ bilayers was varied between 2 and 10, where the first deposited layer was always Fe. The last MgO layer was capped with a 4.2 nm thick Pd layer. The samples were grown in a random order to allow identification of potential systematic errors, for example drift in the growth rate. No systematic drift/change in thickness of the layers was identified.

The structural analysis was performed using x-ray scattering and transmission electron microscopy (TEM). A Philips X-Pert Pro MRD diffractometer (Cu K$_{\alpha } =0.15418$ nm) was used to perform x-ray reflectivity (XRR) and diffraction (XRD) measurements. For the TEM analysis, a sample was prepared in a cross-sectional geometry by conventional sample preparation technique including mechanical polishing, dimple grinding and Ar ion milling. A 200 kV FEI Titan Themis equipped with a probe corrector was operated in scanning TEM (STEM) mode and high angle annular dark field (HAADF) images of the sample were acquired.

The overall magnetic response of the samples was determined using a magneto-optical Kerr effect setup in longitudinal geometry (L-MOKE) with *s*-polarized light, at room temperature. The magnetic field was applied in the plane of the sample (parallel to the optical scattering plane) and the magnetic response was measured parallel to the applied field. The magnetic arrangement of the layers was determined using polarised neutron reflectometry (PNR). The Super ADAM reflectometer^19^ at the Institut Laue-Langevin in Grenoble, France, was used for the PNR measurements (the raw data can be found in Ref.^20^). The neutron wavelength was 0.521 nm and polarizer and analyzer efficiencies were 99.8% and 99.4%, respectively. The consequent spin leakage was compensated for during the data treatment. An electromagnet was used to obtain magnetic fields perpendicular to the scattering plane, which also served as a guide field for the neutrons. The software pySAred^21^ was used to reduce the obtained experimental data. The data was corrected for sample overillumination and normalized by integrated monitor counts (to account for fluctuations in the neutron flux).

## Results and discussion

**Figure 1:**
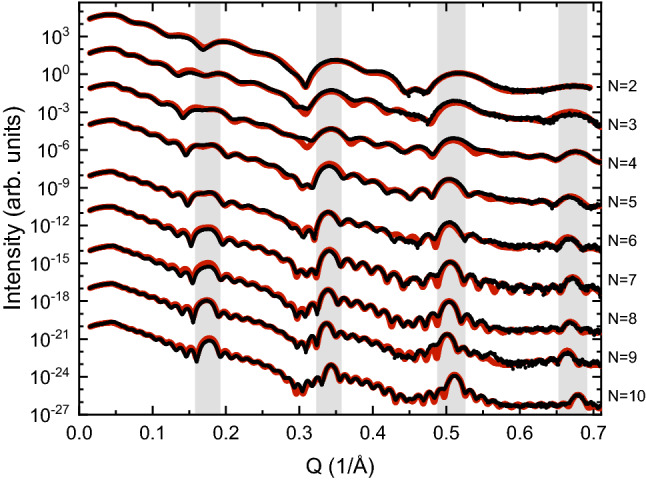
X-ray reflectivity measurements (black dots) and GenX fits (red lines) of [Fe/MgO]$_N$ multilayers. The data is shifted (in intensity) for clarity and the peak positions corresponding to the repeat distance $\lambda$ of the samples, are indicated by light grey shading.

The repeat distance $\lambda$ as well as thickness of the Fe and the MgO layers were determined by x-ray reflectivity (XRR). XRR measurements and fits of the results obtained from [Fe/MgO]$_N$ multilayers are shown in Fig. 1. Clear total thickness oscillations and multilayer peaks are observed up to Q = 0.7 1/Å (2$\theta$ = 10°), consistent with a well defined thickness of the samples. The fitting using GenX^22^ yields an average value of $\lambda$ = 3.72 ± 0.04 nm, reflecting a high degree of reproducibility in the obtained layer thicknesses. A fit of the XRR data for the sample with $N=10$ yields root mean square roughnesses of the top-interface of 0.4 nm for Fe and 0.3 nm for MgO. The x-ray diffraction (XRD) measurements provide limited information on the crystallinity of the samples, resembling the analysis and results described in Ref.^23^ (see supplementary information for details). Since the coupling strength decays exponentially with MgO thickness^8^, even small changes in thickness (0.04 nm) will matter. To ensure control of the uncertainty in the layer thicknesses and quality, samples with identical input parameters were grown at the beginning and end of the series. The control samples were found to be close to identical to the replicated samples (structurally as well as magnetic properties), proving that drifts in the growth rates can be neglected.

**Figure 2:**
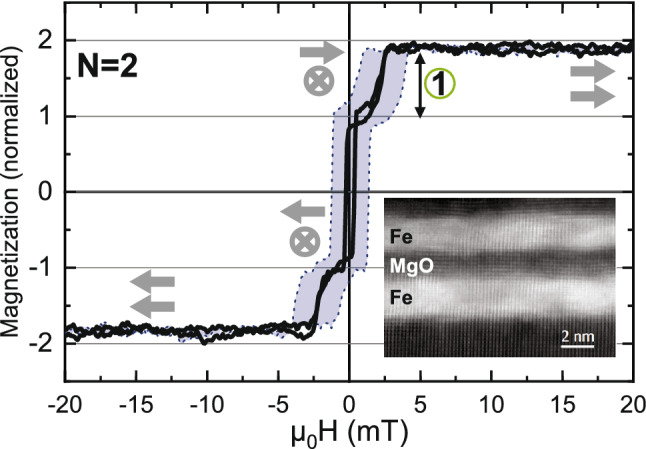
Magnetization measurements along the in-plane easy axis (Fe[100]) of a [Fe/MgO]$_2$ sample. The outline of the blue area marks the measured hysteresis loop whereas in the black curve the coercivity of the Fe layers has been removed. Possible magnetic states are indicated by gray arrows. The layering and the crystallinity of the Fe(001) and MgO(001) lattice is illustrated by a HAADF STEM image of the $N=2$ sample (inset).

**Figure 3:**
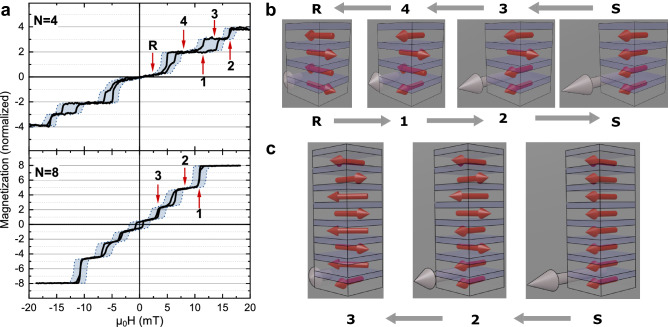
(**a**) Representative results from magnetization measurements along the in-plane easy axis (Fe[100]) of a [Fe/MgO]$_4$ and a [Fe/MgO]$_8$ multilayer. The outline of the blue areas marks the measured hysteresis loops whereas in the black curves the coercivity of the Fe layers has been removed. The arrows and the symbols refer to the external fields used in the neutron experiments. (**b**) Schematic illustration of the field dependent magnetic ordering obtained from fitting the N = 4 PNR data (the angles are rounded off to the nearest easy axis) with S being the saturation. When a field is applied, the outermost layers switch first (R → 1), becoming parallel to the applied field. When the field is decreased, the outermost layers are the last to return to the AFM state (4 → R). (**c**) Schematic illustration of the magnetic arrangement obtained from fitting the N = 8 PNR data. See supplementary information for the PNR data and the values obtained from the fits.

Representative results from magnetization measurements along the Fe [100] (easy) direction of a sample with 2 bilayers is shown in Fig. 2. As seen in the figure, the switching of the Fe layers takes place in discrete steps (sometimes referred to as a digital hysteresis) which can be attributed to a layer-by-layer switching^14,16,24^. The measured hysteresis loop (outline of the blue area) is influenced by the interlayer exchange coupling between Fe layers as well as their coercivity. By removing the coercivity we better reveal the reversibility (or the lack thereof) in the switching of the layers. As seen in the figure, the sample exhibits a switching field (field of the magnetization step) of $\sim$ 1.8 mT and a remanent magnetization of 0.5$M_{s}$, consistent with a 90${^\circ }$ alignment of the two Fe layers in the remanent state. The interlayer coupling between the Fe layers is therefore not sufficiently strong to drive the order to an antiferromagnetic (AFM) state. The 90${^\circ }$ configuration is metastable and enabled by the interplay between the strong four-fold magnetocrystalline anisotropy and the relatively much weaker antiferromagnetic interlayer exchange coupling^8,14,25,26^. The switching field is the field required to overcome the antiferromagnetic coupling and align the two layers. It is therefore directly proportional to the coupling strength. In this particular case, identifying possible magnetic configurations at each step is easy, as there are only two identical layers contributing to the changes in the magnetization. An increase of the number of bilayers (*N*) has a marked effect on the shape of the hysteresis loop as seen in Fig. 3a. The sample with four Fe layers ($N=4$) has three steps in the hysteresis loop (between remanence and saturation) and an approximately nine times higher saturation field ($\sim$ 16 mT) as compared to the sample with $N=2$. The increase in switching field is consistent with an increase in the strength of the interlayer exchange coupling, becoming strong enough to drive the magnetic ordering of the layers into an AFM state at remanence^14^. We note that the number of steps, the switching fields and the shape of the hysteresis loops are changing when changing the number of repeats. The increased complexity with increasing number of repeats therefore calls for additional information on the switching to allow us to uniquely identify the roots of the obtained steps.

We used polarized neutron reflectometry (PNR) to establish the switching order of the layers in two of the samples, $N=4$ and $N=8$. For that, the PNR data acquired at 500 mT (saturation) and the XRR data of the respective samples were fitted simultaneously using GenX^22^, determining the structural parameters of the samples. A more in-depth description of the fitting procedure can be found in Ref.^14^. The hysteresis loop obtained from a [Fe/MgO]$_4$ sample (Fig. 3a) shows steps close to remanence, which are twice as large as the other steps. The GenX^22^ fit of the PNR data (see supplementary information) reveal that the change in magnetization arises from the simultaneous switching of the outermost layers, as illustrated in Fig. 3b. These layers have only half the number of interacting neighbors and their switching field (coupling strength) is therefore the smallest. Increasing the field further leads first to the alignment of layer 3 and finally to the alignment of layer 2 with the external field. Decreasing the field from saturation leads again to the switching of only layer 2, followed by only layer 3 and finally the simultaneous switching of the outermost layers, obtaining full AFM order.

A similar behavior is observed for a [Fe/MgO]$_8$ sample, as illustrated in Fig. 3c. The magnetizations of layer 2, 4 and 6 are switched simultaneously at the highest switching fields (step 1 and 2). The magnetizations of layer 3, 5 and 7 are switched simultaneously at the second highest switching field (step 3). Comparable to the [Fe/MgO]$_4$ sample, different switching fields are observed for the even and odd innermost layers, even though the number of nearest neighbors is identical. Increasing the number of Fe layers to eight also results in a decrease in the highest and the lowest switching fields (as compared to $N=4$), which cannot be rationalized, solely using nearest neighbor interactions. We therefore analyzed the magnetization of the samples along the Fe [110] (hard) directions (see supplementary information). The Fe layers in the different samples were observed to have the same anisotropy field and can therefore be assumed to be in similar strain states, supporting (in combination with the XRR results) that the structural quality of the samples is comparable. Also roughness induced changes of the interlayer exchange coupling can be ruled out as it is constant (see supplementary information). Finally, stray fields arising from the edges of the magnetic layers cannot be responsible for the observed changes with N, as they are negligible for thin films with large lateral dimensions ($\sim$ 1 cm)^26,27^. We therefore need to look closer into the influence of the number on repeats on the switching field of the layers.

**Figure 4:**
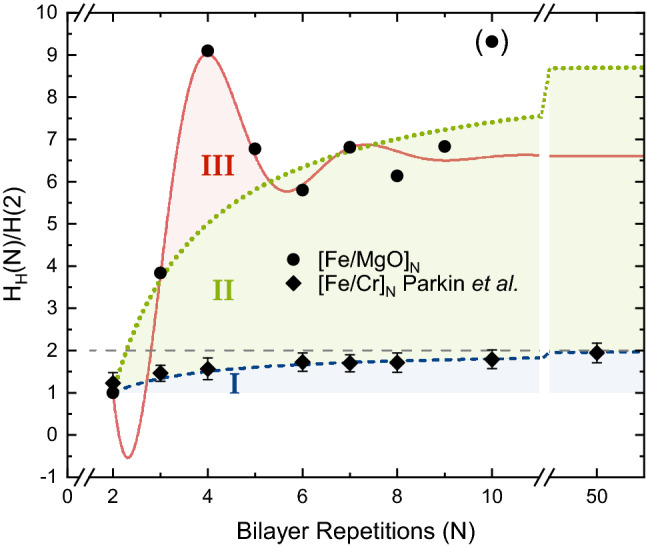
Normalized saturation field, [$H_H(N)/H(2)$], of [Fe/Cr]$_N$ and [Fe/MgO]$_N$(001) samples with different number of bilayer repetitions (*N*). *H*(2) is the switching field of a sample with two Fe layers and $H_H(N)$ is the saturation field of a sample with *N* bilayer repetitions. The dashed, blue line is the fit of the data obtained from Ref.^18^. The dotted, green and solid, red lines are fits of the Fe/MgO(001) multilayers.

In Fig. 4 we plot the saturation field of [Fe/MgO]$_N$ and [Fe/Cr]$_N$ samples as a function of *N*. For [Fe/MgO]$_N$ this value corresponds to the field required to switch the most strongly coupled layers in a discrete manner. The field response of the [Fe/Cr]$_N$ samples is continuous^18^ and does not involve any discrete steps. In an attempt to capture the observed dependence, for both continuous saturation and discrete switching of the layers, we use a simple model based upon the effect of missing neighbors at boundaries on the saturation field of the samples:

1 $$\begin{aligned} H_{H}(N)/H(2) =a\left( 1-\frac{b}{N} \right) \end{aligned}$$

where $H_{H}(N)$ is the saturation field of a sample with *N* repeats, *H*(2) is the saturation field of a reference sample with 2 Fe layers, *a* defines the relative strength of the coupling in a multilayer with large number of repetitions (*N*) as compared to a sample with *N* = 2, while *b* defines the ratio of the coupling in the small and large repetition limit. The observed changes in the coupling strength in Fe/Cr multilayers^18^ are fully reproduced by Eq. (1) with $a=2$ and $b=1$, consistent with interactions restricted to nearest neighboring Fe layers. Direct exchange between Fe and Cr at the interfaces, in combination with the Cr-Cr coupling in the Cr layers are the cause of coupling between the Fe layers. The coupling between the Fe layers will therefore always appear nearest neighbor like in Fe/Cr multilayers. The same model with $a=9$ and $b=16/9$ does not captures the changes in the saturation field (highest switching field) of the epitaxial [Fe/MgO]$_N$ multilayers (region II in Fig. 4) as well and we notice clear outliers at $N=4$ and $N=10$. These outliers cannot be due to differences in thickness of the Fe and MgO layers in the different samples, which calls for an alternative explanation.

Oscillatory interlayer coupling as a function of the magnetic layer thickness has been demonstrated in single, MgO-based magnetic tunnel junctions^28^ which is rationalized in terms of interference of Bloch waves, arising from multiple reflections of electrons within the metal layers at the metal/insulator interfaces^29^. A similar effect is conceivable in superlattices with different number of repeats. Assuming that the coherence of the electron wave function is conserved throughout the multilayer, one can expect an interference of partially reflected waves from all interfaces and a full reflection at the outermost layers. In this case, the total extension of the multilayer would result in oscillations in the coupling strength. Indeed, the changes in coupling strength of epitaxial [Fe/MgO]$_N$ multilayers can be reasonably well described by an exponentially decaying sine function with a periodicity of *N* = 3.3, or 12.2 nm (region III in Fig. 4). Hence, an electron phase coherence length of at least 12.2 nm is needed to observe the reported oscillation period, in line with a previously reported value of 12.6 nm in Fe/oxide heterostructures^30^. These oscillations would be observed in the coupling strength of the outermost Fe layers (lowest switching fields) as well as the inner Fe layers (highest switching fields). We therefore look into the changes in the switching of all the layers in the samples with different number of repeats.

**Figure 5:**
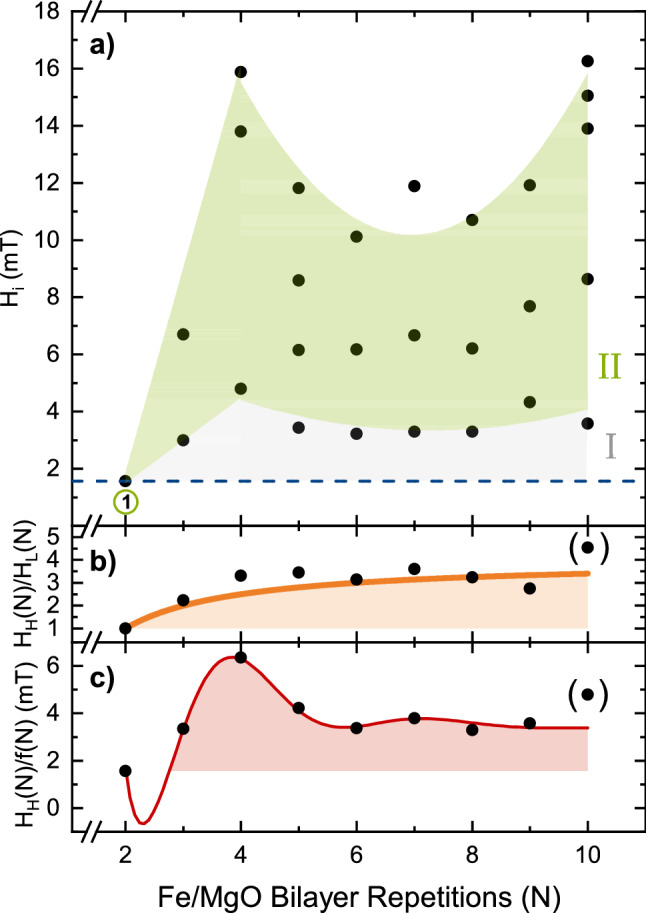
(**a**) Switching fields (ascending hysteresis loop) of the i-th step as a function of bilayer repetitions. Switching field of samples with $N=2$ is labeled corresponding to the labeling of the magnetization steps in Fig. 2. The outermost Fe layers switch at the lowest fields due to the weaker interlayer coupling arising from missing neighbors. (**b**) Normalized saturation fields, [$H_H(N)/H_L(N)$], and fit *f*(*N*) (orange line) of the data shown above with $H_H(N)$ being the highest and $H_L(N)$ being the lowest switching field of the respective sample. (**c**) $H_H(N)$ divided by the fit *f*(*N*) shown above. The fit (red line) corresponds to $f(\lambda )$, as described in the text below.

The identified (discrete) switching fields with increasing field, for samples with 2 ≤ *N* ≤ 10, are plotted in Fig. 5a. For $N=2$, increasing the field from zero to saturation results in one discrete step in the hysteresis (see Fig 2). In this case, the switching field is proportional to the coupling strength between the two adjacent Fe layers, both having only one neighbor. When increasing the number of repeats to 3, two of the layers have one nearest and one second nearest neighbor while the center layer has two nearest neighbors and no second nearest neighbors. Consequently, two switching fields are observed. The lower switching field corresponds to the magnetization reversal of the outermost layers and is directly proportional to their coupling strength. If the interaction is restricted to nearest neighbors only and the coupling strength of the layers would be independent of *N*, the switching field of the outermost layers would be the same for all samples. As seen in Fig. 5a, we observe a significant increase in the coupling strength of the outermost layers (highlighted as region I) in line with the oscillatory behavior of the strongest coupled layers (Fig. 4). Hence, two contributions of the interlayer exchange coupling of [Fe/MgO(001)]$_N$ superlattices as a function of *N* are apparent and can be described by $H_{H}(N) = f(N)\cdot f(\lambda )$ where $H_{H}(N)$ is the saturation field of a sample with *N* repeats, *f*(*N*) is the function shown in Eq. (1) (corresponding to beyond nearest neighbor interactions^14^) and $f(\lambda )$ is a function to capture the impact of the total thickness of the sample on the saturation field. These contributions can be separated. For example, the effect from the sample thickness can be removed by normalizing the highest switching field (strongest coupled layers) of each sample to the lowest switching field (outermost layers) of the respective sample: $H_{H}(N)/H_{L}(N)=a\left( 1-\frac{b}{N} \right)$ where $H_{L}(N)$ is the lowest switching field and $H_{H}(N)$ corresponds to the highest switching field, as shown in Fig. 5b. The fit (with $a=4$ and $b=3/2$) describes the changes in the interlayer exchange coupling arising solely from the changes in the number of nearest neighbors. This contribution is determined to be four times larger in samples with large *N* as compared to $N=2$, using the model described above. The second contribution is revealed by dividing the highest switching fields $H_{H}(N)$ by the fit of the first contribution *f*(*N*), as shown in Fig. 5c. We use the same function shown as region III in Fig. 4 (divided by the first contribution *f*(*N*) and multiplied by H(2)) to fit the resulting data. The contribution to $f(\lambda )$ is therefore argued to arise solely from the samples’ total thickness. The thickness of a sample with fixed *N* can still be continuously chosen, e.g., through the selection of the Fe layer thicknesses. This can give rise to alias effects (similar to those observed in RKKY like coupled samples) resulting in a non-discrete oscillation period of *N* = 3.3.

## Conclusions

The saturation fields of antiferromagnetically coupled [Fe/MgO(001)]$_N$ superlattices depend strongly on the total number of repetitions (*N*). The observed changes are argued to stem from two sources: beyond nearest neighbor interactions^14^ as well as changes in the coupling strength between layers due to the total extension of the superlattices. Our observations are consistent with non-linear contributions to the interlayer exchange coupling, possibly arising from the influence of the outer boundaries of the samples on the previously inferred tunnelling mediated coupling^9–11^. Support for the interpretation is obtained from the oscillatory and increasing strength of the saturation of the outermost Fe layers with increasing *N*. The results challenge our current understanding of interlayer exchange coupling in metal-oxide heterostructures, with potential impact on emerging spin-based technologies.

## Supplementary Information

Supplementary Information.
