# Supplementary material for: The impact of number of repeats N on the interlayer exchange in [Fe/MgO]N(001) superlattices
Source: Sci Rep. 2021 Jan 21;11:1942. doi: 10.1038/s41598-021-81441-y (PMC7820583; doi:10.1038/s41598-021-81441-y)
Supplement: Supplementary file 1 — Supplementary Information. [file 41598_2021_81441_MOESM1_ESM.pdf]

SUPPLEMENTARY INFORMATION

The impact of number of repeats *N* on the interlayer exchange in $[Fe/MgO]_N(001)$ superlattices

Tobias Warnatz[1], Fridrik Magnus[2], Nanny Strandqvist[1], Sarah Sanz[1,a], Hasan Ali[3], Klaus Leifer[3], Alexei Vorobiev[1] and Björgvin Hjövarsson[1,*]

[1]Department of Physics and Astronomy, Uppsala University, Box 516, SE-75120 Uppsala, Sweden
[2]Science Institute, University of Iceland, Dunhaga 3, IS-107 Reykjavik, Iceland
[3]Department of Materials Science and Engineering, Uppsala University, Box 534, SE-75121 Uppsala, Sweden
[a]Present address: University of Konstanz, Constance, Germany
*bjorgvin.hjorvarsson@physics.uu.se

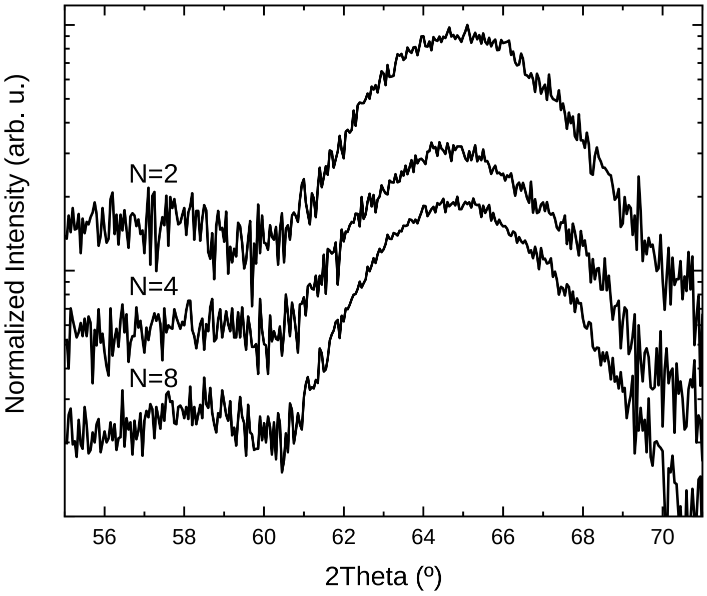

Supplementary Fig. 1 X-ray diffraction measurements of [Fe/MgO]$_N$ superlattices illustrating similar full-width-half-maxima and peak positions of all three samples. The increased number of repeats leads to a more pronounced Laue oscillation (around 58.4° 2Theta) due to an increased signal to noise ratio with increasing N. The absence of superlattice peaks is attributed to an interplay of the incommensurate lattice constants of the Fe and MgO layers and their thickness variation.

Supplementary Tab. 1 Roughness values obtained from the XRR fits (red lines) shown in Fig. 1.

**N**	Root mean square roughness **Fe** top-interface	Root mean square roughness **MgO** top-interface
2	0.4 nm	0.2 nm
3	0.3 nm	0.2 nm
4	0.5 nm	0.3 nm
5	0.8 nm	0.3 nm
6	0.4 nm	0.3 nm
7	0.5 nm	0.3 nm
8	0.5 nm	0.3 nm
9	0.4 nm	0.3 nm
10	0.4 nm	0.3 nm

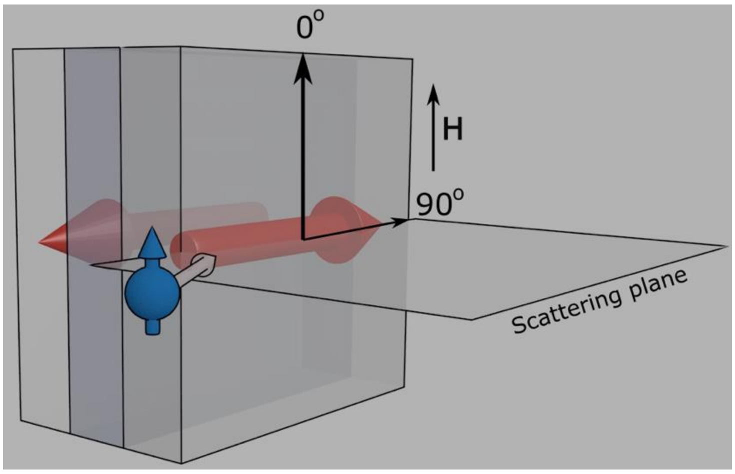

Supplementary Fig. 2 Scattering geometry of PNR measurements. Neutrons (dark blue) are initially polarized along the 0° axis (parallel to the applied field H). The magnetization direction of Fe layers is indicated by red arrows. A MgO layer is illustrated in light blue.

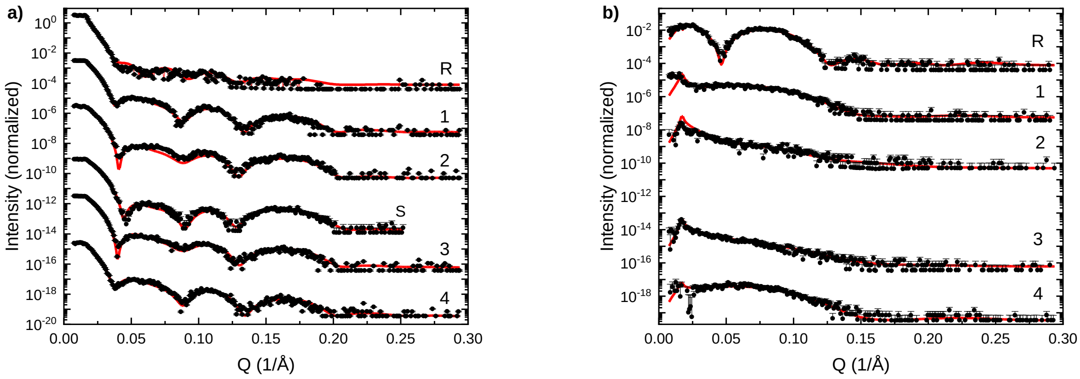

Supplementary Fig. 3 Non-spin-flip (a) and spin-flip (b) polarized neutron reflectometry measurements (black dots) and GenX fits (red lines) of a [Fe/MgO]$_4$ multilayer. The data is shifted (in intensity) for clarity. The measurements correspond to different external fields, as indicated in Fig. 3a.

Supplementary Tab. 2 Magnetization angles of Fe layers in a [Fe/MgO]$_4$ multilayer, as determined from fitting the PNR data (Supplementary Fig. 3) taken at the field values indicated in Fig. 3a. 0° is parallel to the applied field direction (Supplementary Fig. 2). The angles corresponding to switched layers (biggest difference to previous position) have been highlighted in bold.

Fe Layer	R	1	2	S	3	4
1	93°	**-4°**	2°	0°	7°	1°
2	-76°	-93°	-75°	**0°**	**-86°**	-95°
3	102°	70°	**3°**	0°	11°	**75°**
4	-75°	**-20°**	-5°	0°	0°	-13°

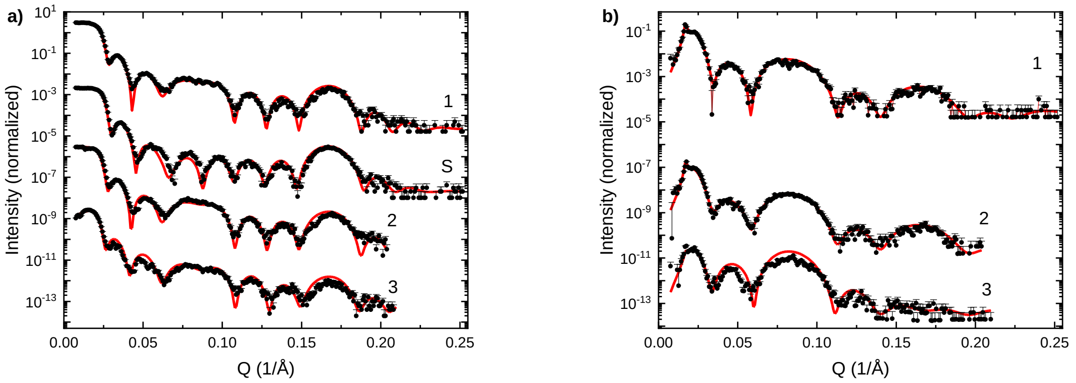

Supplementary Fig. 4 Non-spin-flip (a) and spin-flip (b) polarized neutron reflectometry measurements (black dots) and GenX fits (red lines) of a [Fe/MgO]$_8$ multilayer. The data is shifted (in intensity) for clarity. The measurements correspond to different external fields, as indicated in Fig. 3a.

Supplementary Tab. 3 Magnetization angles of Fe layers in a [Fe/MgO]$_8$ multilayer, as determined from fitting the PNR data (Supplementary Fig. 4) taken at the field values indicated in Fig. 3a. 0° is parallel to the applied field direction (Supplementary Fig. 2). The angles corresponding to switched layers (biggest difference to previous position) have been highlighted in bold.

Fe Layer	1	S	2	3
1	12°	0°	11°	6°
2	-77°	**0°**	**-91°**	-100°
3	8°	0°	21°	**59°**
4	-82°	**0°**	**-91°**	-99°
5	0°	0°	-2°	**41°**
6	-85°	**0°**	**-91°**	-96°
7	-9°	0°	0°	**57°**
8	2°	0°	0°	-5°

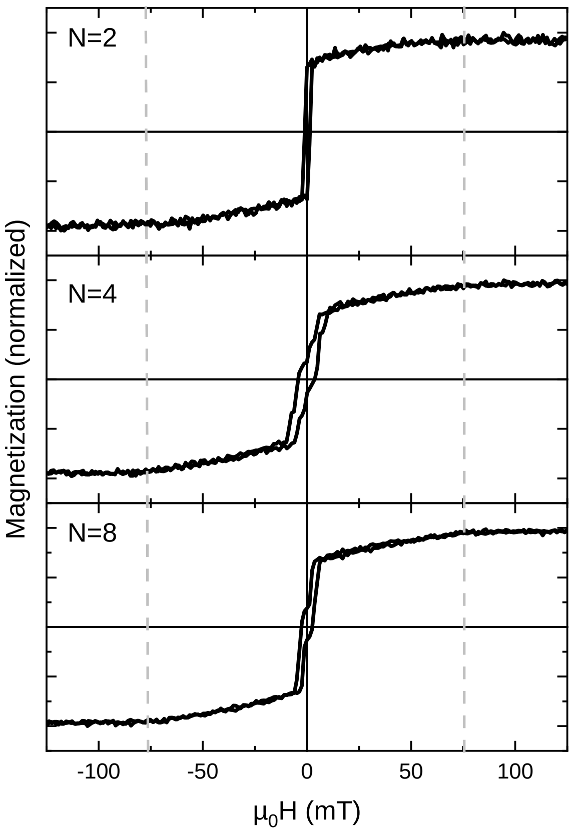

Supplementary Fig. 5 Complementary magnetization measurements along the in-plane hard axis (Fe[110]) of the samples presented in Fig. 2 and 3a. The dashed grey lines illustrate identical saturation fields of all three samples.

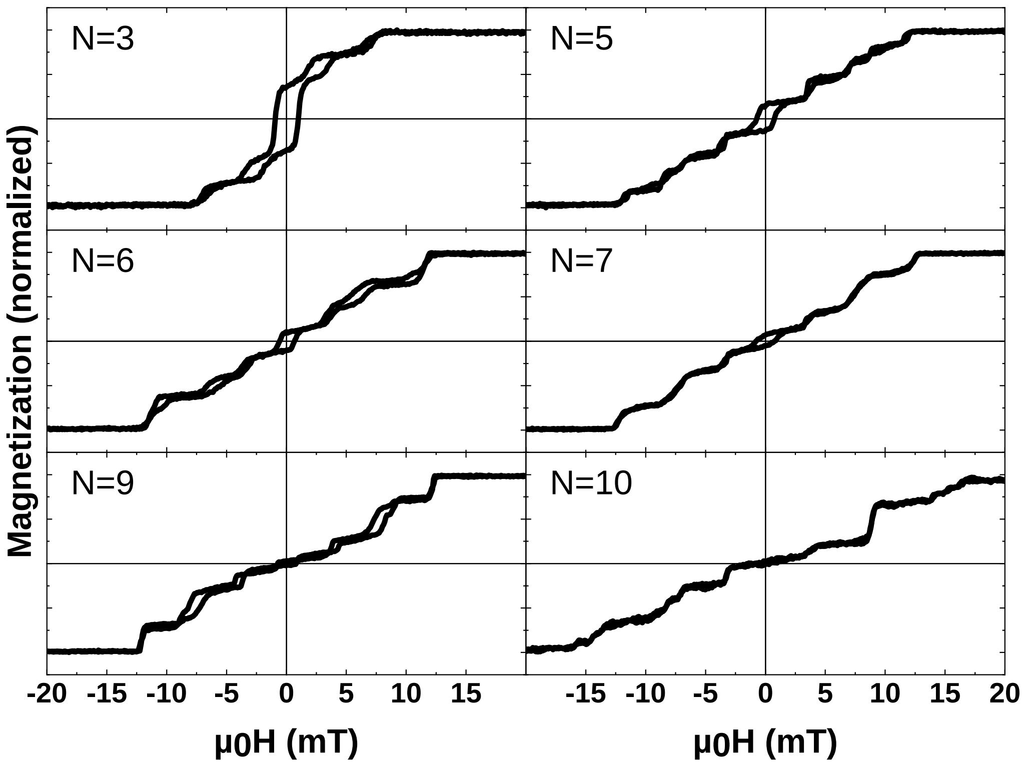

Supplementary Fig. 6 Magnetization measurements along the in-plane easy axis (Fe[100]) of samples with different bilayer repetitions N. The coercivity of the Fe layers has been removed to highlight the switching fields.
